# Focused Pulse High-frequency Chest Wall Oscillation (FP-HFCWO) for Mucus Management in COPD (Chronic Obstructive Pulmonary Disease) and Asthma: A Single-center Cohort Study

**DOI:** 10.2174/0118743064391693250627115047

**Published:** 2025-07-03

**Authors:** Pier-Valerio Mari, Angelo Coppola, Carriera Lorenzo, Macagno Francesco

**Affiliations:** 1 San Carlo di Nancy Hospital, Rome (IT). Italy; 2 San Filippo Neri Hospital, Rome (IT), Italy; 3 Università Cattolica del Sacro Cuore, Rome, Italy; 4 Fondazione Policlinico Universitario A. Gemelli, IRCCS, Rome, Italy

**Keywords:** Chronic mucus hypersecretion, COPD, Asthma, FP-HFCWO, Respin 11, Exacerbations, Treatable trait

## Abstract

**Introduction:**

Mucus hypersecretion is a significant clinical challenge in patients with Chronic Obstructive Pulmonary Disease (COPD) and asthma, often contributing to poor disease control and frequent exacerbations despite maximal pharmacological therapy. Focused Pulse High-Frequency Chest Wall Oscillation (FP-HFCWO) therapy has been proposed as an adjunctive treatment to enhance mucus clearance and improve clinical outcomes. This retrospective cohort study aimed to assess the impact of FP-HFCWO therapy using the Respin 11 device in patients with COPD and asthma presenting with persistent mucus hypersecretion and inadequate disease control.

**Methods:**

A retrospective, single-center analysis was conducted on patients with COPD or asthma attending the pulmonary outpatient clinic at San Carlo di Nancy Hospital in Rome from September 2023 to January 2025. Eligible patients were those receiving maximal inhalation therapy, daily mucolytics, and presenting with mucus hypersecretion, frequent exacerbations or radiological small airways impairment such as tree-in-bud or mucus plug. FP-HFCWO therapy was prescribed for 20 minutes daily, and clinical outcomes were evaluated using the COPD Assessment Test (CAT) and Asthma Control Test (ACT) scores. The effect of FP-HFCWO on CAT/ACT changes and moderate-to-severe exacerbations was evaluated.

**Results:**

A total of 27 patients were included (COPD: n=17, asthma: n=10). The mean age was 74.2 years, with 78.0 for COPD and 67.8 for asthma. Baseline spirometry showed greater obstruction in COPD (FEV_1_% predicted: 64.7%) compared to asthma (78.2%). Both groups share a significant small airway involvement on High-Resolution Computed Tomography (HRCT) and a smoking habit. FP-HFCWO significantly improved the clinical burden, CAT scores decreased by a mean of 7.5 points (*p*<0.001) in COPD, while ACT scores improved by 7.5 points (*p*<0.001) in asthma. The number of moderate-to-severe exacerbations was reduced by 66.6% in asthma (Δ-1.20 events) and by 73.0% in COPD (Δ-1.11 events), both statistically significant (*p*<0.001).

**Discussion:**

These findings suggest that FP-HFCWO therapy may serve as a valuable adjunct to standard care in obstructive airway diseases. The observed improvements in symptom scores and reduction in exacerbations support its clinical relevance. Despite the retrospective design and limited sample size, the consistency of benefit across both COPD and asthma groups is noteworthy. This therapy could be considered in selected patients with chronic mucus hypersecretion and poor disease control. Further prospective studies are needed to confirm these promising results and define optimal patient selection criteria.

**Conclusion:**

FP-HFCWO therapy (Respin 11, VitalAire®) demonstrated significant clinical benefits in patients with Chronic Mucus Hypersecretion (CMH) and poor disease control, leading to improved symptom burden and reduced exacerbation frequency in both COPD and asthma populations. These findings support the use of FP-HFCWO as an effective adjunctive therapy in COPD and asthma. However, given the study's limitations, including its retrospective design, small sample size, single-center setting, and short follow-up duration, further large-scale prospective studies are warranted to validate these results and assess long-term benefits.

## 1 INTRODUCTION

Chronic Mucus Hypersecretion (CMH) is a hallmark feature of obstructive pulmonary diseases such as Chronic Obstructive Pulmonary Disease (COPD) and asthma [1, 2]. It plays a crucial role not only in airflow limitation, particularly in peripheral airways, but also in fostering an environment conducive to disease exacerbations [3]. This occurs through multiple mechanisms, including the induction of regional hypoxia and the promotion of pulmonary microbiota dysbiosis, which can contribute to a pro-inflammatory state [4, 5].

The presence of airway-occluding mucus plugs in COPD patients is significant, with prevalence estimates ranging from 41% to 70% [6]. These mucus plugs are associated with worsened pulmonary function, as evidenced by spirometry and imaging assessments [7]. Notably, recent evidence has shown that the presence of airway-occluding mucus plugs in patients with COPD is independently associated with increased mortality risk, underscoring the potential impact of mucus clearance strategies in modifying long-term outcomes [7, 8]. Thus, addressing mucus clearance in these patients could therefore represent a pivotal therapeutic target.

In asthma, CMH is similarly linked to worse clinical outcomes, including increased dyspnea, reduced disease control, and higher exacerbation frequency [9]. Excess mucus production in asthma contributes to airway obstruction and can lead to life-threatening episodes in severe cases [9]. Recent longitudinal studies have shown that mucus plugs in asthma are not only persistent over time but also correlate with airflow limitation, reinforcing their relevance as a therapeutic target [10].

While the clinical significance of CMH is well established, evidence regarding the efficacy of mucolytic agents in COPD and asthma remains inconclusive. Similarly, the role of airway clearance devices, such as Positive Expiratory Pressure (PEP) therapy and High-Frequency Chest Wall Oscillation (HFCWO) [6], in the management of obstructive lung diseases has not been definitively determined. As a result, the optimal technique for mucus clearance in these patients has yet to be standardized.

A novel variation of HFCWO, termed “focused pulse (FP), has recently garnered attention due to its ease of use and applicability even in patients with limited mobility. This technique delivers targeted percussive forces within the bronchial tree, potentially enhancing mucus clearance efficacy [11].

Initial clinical studies have explored the efficacy of FP-HFCWO in COPD and other obstructive pulmonary conditions [12, 13]. Findings indicate improvements in both quality-of-life indices and hospital length of stay during acute exacerbations. Additionally, preliminary data suggest that FP-HFCWO therapy may facilitate sputum reduction and contribute to better management of acute COPD and asthma exacerbations. This raises the clinically relevant question of whether prolonged use of this device could reduce long-term exacerbation rates.

Despite promising findings, current literature does not adequately address whether patients included in these studies were receiving optimized pharmacological therapy, including mucolytic treatment [14]. Additionally, there are limited data on the radiological characterization of mucus burden and the long-term follow-up. These gaps highlight the necessity for further high-quality clinical studies to establish the definitive role of airway clearance devices in obstructive lung disease management.

To address these knowledge gaps, FP-HFCWO therapy (Respin 11, VitalAire®) was evaluated in our clinical center. This study specifically targets patients already on maximal pharmacological therapy, including optimal mucolytic treatment, to assess the incremental benefit of adding this device to their regimen. This investigation aimed to elucidate the role of FP-HFCWO in enhancing mucus clearance and improving clinical outcomes in patients with obstructive lung diseases.

## 2 METHODS

This study was a retrospective observational cohort study conducted at the Pulmonology Outpatient Clinic of San Carlo di Nancy Hospital (Rome, Italy) between September 2023 and January 2025. The analysis included patients with a confirmed diagnosis of COPD or asthma who exhibited chronic mucus hypersecretion and persistent symptoms despite maximal pharmacological treatment. Patients were identified through electronic medical records and outpatient charts. The intervention of FP-HFCWO with Respin 11 was prescribed based on clinical indication, and outcomes were evaluated by comparing symptom scores *via* CAT/ACT and exacerbation frequency before and after the intervention, using a within-subject design. No randomization or control group was used. As a retrospective study, informed consent was waived in accordance with institutional policies.

### 2.1 Patient Selection

**Fig. (1):**
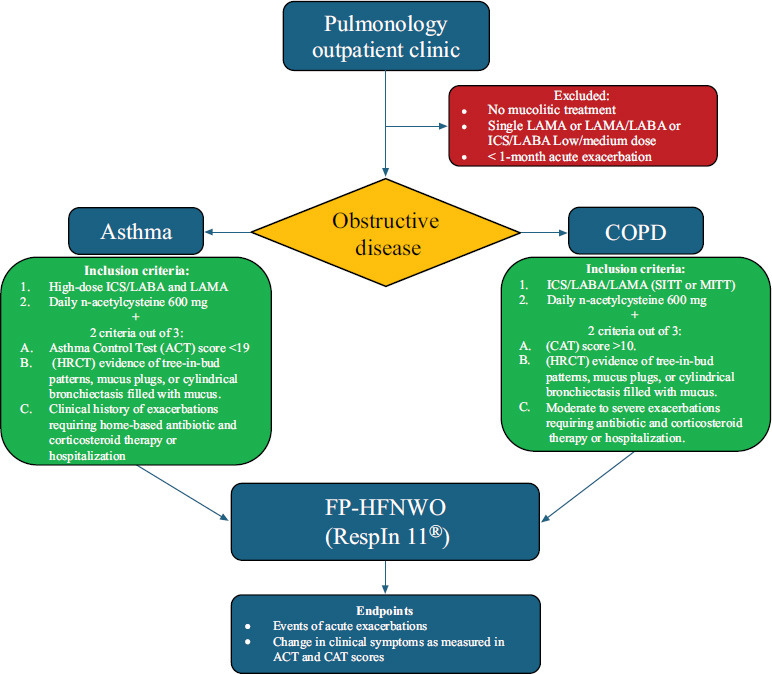
Flowchart of the selection process for patients who were eligible to start treatment with FP-HFCWO

Patients included in the study had obstructive respiratory diseases with features of Chronic Mucus Hypersecretion (CMH) and were stratified into two groups, asthma and COPD (Fig. **1**). The inclusion criteria for FP-HFCWO therapy comprised significant mucus hypersecretion, poor symptom control, and frequent exacerbations despite the best medical management. Regarding the exclusion criteria, patients who refused the device, patients with < 1-month acute exacerbation, those who did not receive daily mucolytic therapy, and those without triple treatment in COPD or GINA Step 5 treatment in asthma were excluded. This retrospective cohort study included a total of 27 patients (17 with COPD and 10 with asthma) consecutively enrolled between September 2023 and January 2025 at the Pulmonology Outpatient Clinic of San Carlo di Nancy Hospital, Rome. The mean age of the population was 74.2 years (SD 13.1), with an age range of 37 to 91 years. The sample included both male and female subjects, with 59% male (n=16) and 41% female (n=11) representation.

### 2.2 COPD

Patients with COPD considered for FP-HFCWO therapy were those receiving maximal pharmacological treatment, including Inhaled Corticosteroids (ICS), Long-Acting Beta-Agonists (LABA), and Long-Acting Muscarinic Antagonists (LAMA), along with daily mucolytic therapy (N-acetylcysteine 600 mg). Eligibility required the presence of at least two of the following characteristics:

- A COPD Assessment Test (CAT) score >10.
- High-Resolution Computed Tomography (HRCT) evidence of tree-in-bud patterns, mucus plugs, or cylindrical bronchiectasis filled with mucus.
- A history of moderate to severe exacerbations requiring antibiotic and corticosteroid therapy, hospitalization, oxygen treatment or mechanical ventilation.

### 2.3 Asthma

Asthmatic patients eligible for FP-HFCWO therapy were those undergoing maximal pharmacological treatment, including high-dose ICS/LABA and LAMA, alongside daily mucolytic therapy with N-acetylcysteine (NAC) 600 mg. Eligibility required the presence of at least two of the following criteria:

- An Asthma Control Test (ACT) score <19, indicating partially controlled asthma.
- HRCT findings of tree-in-bud patterns, mucus plugs, or cylindrical bronchiectasis filled with mucus.
- Clinical history of requiring antibiotic and corticosteroid therapy, hospitalization, oxygen treatment or mechanical ventilation.

### 2.4 FP-HFCWO

Eligible patients were prescribed and provided with the FP-HFCWO therapy (Respin 11, VitalAire®) set to the “FIRM” mode. Patients were instructed to use the device for 20 minutes daily, every day.

#### 2.4.1 Endpoints and Outcome Measures

The study analyzed the impact of the FP-HFCWO therapy by assessing the following endpoints:

- Number of acute exacerbations during the observation and follow-up period.
- Changes in clinical symptoms as measured by variations in ACT and CAT scores.

The data collected aimed to provide insights into the efficacy of the Respin 11 device in improving clinical and radiological outcomes in patients with chronic mucus hypersecretion and persistent respiratory symptoms despite optimal pharmacological treatment. Informed consent was waived by the institutional review board.

### 2.5 Statistics

All statistical analyses were conducted using the latest version of Stata (StataCorp. 2023. Stata Statistical Software. College Station, TX: StataCorp LLC). Continuous variables were expressed as means and standard deviations, while categorical variables were reported as counts and percentages. For pre- and post-intervention comparisons of CAT and ACT scores, as well as the number of exacerbations, paired t-tests were used, given the approximately normal distribution of the data and the within-subject design. Considering the exploratory and hypothesis-generating nature of this real-world analysis, no formal *a priori* power calculation was performed. However, post hoc analysis showed that the observed differences in CAT and ACT scores, as well as exacerbation rates, were statistically significant with *p*-values < 0.001, indicating that the sample size was adequate to detect clinically meaningful changes within this cohort. The study was not powered to detect differences between subgroups, and results should be interpreted in the context of an exploratory design.

### 2.6 Ethical Considerations

Considering the retrospective nature of the analysis and the anonymization of patient data, informed consent was waived in accordance with institutional policy and applicable national regulations. Given the retrospective nature of the study and the use of fully anonymized data, formal ethical approval and informed consent were not deemed applicable, in accordance with the general institutional practice at the San Carlo Di Nancy Hospital And The Guidance Framework Of The Territorial Ethics Committee Lazio.

## 3 RESULTS

The retrospective analysis conducted since October 2023 of our patients attending the pneumology clinic at San Carlo di Nancy Hospital, depicts 27 patients undergoing treatment with FP-HFCWO. Seventeen were diagnosed with COPD and 10 with asthma. All of the evaluated patients were on maximal therapy with LABA/LAMA/ICS for COPD or high-dose ICS/LABA for bronchial asthma. Both groups received mucolytic therapy with NAC 600 mg daily. Table **1** describes the main characteristics of the dataset.

The dataset included a total of 27 patients with a mean age of 74.2 years (SD 13.1, range 37–91), highlighting a predominance of elderly participants. The median duration of follow-up was 7 months (mean 8 months, SD 5.1, range 1–15 months), indicating substantial variability in treatment duration across individuals. The spirometric data revealed a fixed ratio (FEV1/FVC) of 0.67± 0.14 (mean±S; range 0.39–0.94) along with a mean FVC% of 88.9% (SD 13.5, range 48–110%), and a mean FEF25-75% of 47.2% (SD 14.4, range 17–67%), reflecting moderate-to-severe small airway involvement in a notable proportion of patients while the baseline FEV1% averaged 69.7% (SD 18.1, range 31–102%). Active smoking was prevalent in 55% of the dataset, while 29% were former smokers and 16% never smoked.

The clinical questionnaire scores showed a mean CAT baseline of 23.2 points (SD 6.0, range 14–34), reflecting a significant symptom burden in the COPD group, with post-treatment scores improving to a mean of 15.7 points (SD 3.7). Conversely, in patients affected by asthma, ACT scores averaged 15.5 at baseline (SD 2.0, range 11–18) and 22.3 post-treatment (SD 2.0, range 20–24), suggesting substantial improvement.

Regarding exacerbations, the mean number of events in the 12 months before FP-HFCWO therapy was 1.63 (SD 0.93, range 0–3), while follow-up showed a mean of 0.48 events (SD 0.51, range 0–1), reflecting a significant reduction in respiratory episodes.

Moreover, 51.9% of patients demonstrated evidence of small airway involvement on HRCT, such as tree-in-bud or mucus plug, confirming structural abnormalities in over half of the cohort.

The analysis of the two subsets, COPD and asthma, highlights that the characteristics of the dataset are heterogeneous, even though they share common features, such as small airway involvement or advanced age.

The comparison between COPD and asthma patients highlights key differences across clinical variables. COPD patients had a higher mean age (differential: +10.2 years, *p*-value: 0.103) and a shorter time on Respin 11 (differential: -2.12 months, *p*-value: 0.348). The mean fixed ratio was significantly lower in the COPD group, with a differential of -22.0% (*p*-value: 0.001) though the severity of the obstructive pattern is similar with a FEV1% in COPD (differential: -13.4%, *p*-value: 0.059) and the same FVC% (differential: -2.62%, *p*-value: 0.665). The FEF25-75%, a biomarker of small airway disease, was also reduced in both asthma and COPD.

The execution of the FP-HFCWO therapy had a significant clinical benefit, as shown in Table **2** and Fig. (**2**) in the COPD/Asthma subgroups.

In COPD patients, the baseline CAT score was 23.2 with a standard deviation of 6.0 and improved by 7.52 with a standard deviation of 3.14 and a *p*-value of 0.001, reaching 15.7. Moreover, in asthma, the execution of the FP-HFCWO had a significant clinical benefit, as shown in Fig. (**2**) within the COPD/Asthma subgroups. In asthma patients, the baseline ACT score was 15.5 with a standard deviation of 1.95, and during treatment with Respin 11, it increased to 23.0 with a standard deviation of 2.544 and a *p*-value of 0.0001. Therefore, the Respin 11 device significantly improved this score in the population affected by bronchial asthma.

The analysis of exacerbations before and after FP-HFCWO therapy (Respin 11, VitalAire®) observed a significant reduction in both COPD and asthma patients. Specifically, COPD patients showed an absolute reduction of 1.11 exacerbations (mean baseline: 1.529, post-treatment mean: 0.412), while asthma patients exhibited an absolute reduction of 1.20 exacerbations (mean baseline: 1.80, post-treatment mean: 0.60). The results were statistically significant, with a *p*-value of <0.001 in both groups. These findings are shown in Fig. (**3**) and indicate a notable clinical benefit in reducing exacerbation frequency following treatment. In addition to the findings, the role of mucus plugs and tree-in-bud patterns—indicative of small airway involvement—appears to be clinically significant. Although not a mandatory inclusion criterion, such radiological features were present in half of the studied population (8/17 in COPD and 5/10 in asthma). The subgroup analysis of patients with HRCT-documented small airway alterations yielded noteworthy results, demonstrating a statistically significant improvement in obstructive disease scores (ΔCAT: -7.0 ± 1.10, *p*=0.0004; ΔACT: +7.2 ± 1.59, *p*=0.0107), as well as a substantial reduction in moderate-to-severe exacerbations of 1.15 ± 0.19 (*p*=0.0001). These preliminary findings suggest that small airway disease, as visualized on thoracic imaging, may represent an important treatable trait and warrant further investigation in future prospective clinical trials.

## 4 DISCUSSION

In this retrospective analysis, patients diagnosed with chronic obstructive pulmonary disease (COPD) and bronchial asthma were included who also exhibited significant mucus production and lacked clinical stability despite receiving both maximal inhalation therapy and mucolytic treatment. The evaluation of this specific Treatable Trait yielded intriguing results, particularly regarding the therapeutic effects of FP-HFCWO therapy (Respin 11, VitalAire®).

First and foremost, it is important to highlight the demographic differences between the two studied populations. The COPD group had a higher mean age of 78 years compared to the asthma group, with 67.8 years. This discrepancy is likely due to the progressive decline in airway clearance mechanisms with aging, which leads to increased mucus accumulation and, consequently, poses a relevant factor to consider in clinical management. The two groups also differed in terms of functional impairment, with COPD patients exhibiting more pronounced airflow obstruction (mean FEV_1_% predicted: 64.7%) compared to the bronchial asthma group (mean FEV_1_% predicted: 78.2%).

Despite these demographic and functional differences, some striking similarities emerged between the two populations. Specifically, a comparable prevalence of active cigarette smoking was observed with 52.9% in COPD *vs*. 60% in the asthma groups. More importantly, both groups demonstrated peripheral airway involvement as assessed by High-Resolution Computed Tomography (HRCT). Imaging findings consistently revealed diffuse micronodules, tree-in-bud patterns, and mucus plugs, underscoring that persistent mucus accumulation and peripheral airway inflammation serve as key contributors to the lack of clinical stability and poor disease control, even under maximal inhalation and mucolytic therapy. The implementation of daily treatment with Respin 11—performed for 20 minutes per day—led to a marked clinical improvement in both groups, as assessed by validated questionnaires: the COPD Assessment Test (CAT) in COPD patients and the Asthma Control Test (ACT) in asthma patients. The FP-HFCWO therapy significantly enhanced these scores, primarily due to improved mucus clearance and increased sputum expectoration induced by the device. Specifically, the CAT score improved by approximately 7.5 points, while the ACT score increased by a similar margin of 7.5 points, demonstrating a substantial impact on disease burden and patient-reported outcomes. Given the absence of a control group, it is acknowledged that improvements in CAT and ACT scores may be partially influenced by placebo effects or regression to the mean. However, the magnitude of score changes exceeded established minimal clinically important differences, and the simultaneous reduction in exacerbation rates—an objective marker—supports a meaningful clinical benefit.

Moreover, a clinically relevant reduction in the frequency of moderate-to-severe exacerbations was observed. The number of exacerbations decreased by an absolute value of 1.20 episodes (-66.6%) in the asthma group and by 1.11 episodes (-73.0%) in the COPD group. This reduction in exacerbation frequency is of considerable importance, as it contributes to preserving patients’ muscle mass, thereby mitigating the risk of deconditioning, deterioration in quality of life, and overall decline in life expectancy. These findings are consistent with previous reports [12], which demonstrated significant clinical improvements with FP-HFCWO therapy in COPD patients. However, This study also included patients with severe asthma, providing early evidence that FP-HFCWO may confer similar benefits in this population. The observed improvements in ACT scores and reduction in exacerbation frequency highlight the potential of airway clearance therapy as a relevant adjunctive intervention in asthma management. This area remains underexplored in current literature.

These findings suggest that FP-HFCWO therapy represents a valuable adjunctive strategy for improving clinical stability in patients with mucus-related obstructive lung disease. Future prospective studies may further elucidate its long-term impact on disease progression and healthcare utilization.

## 5 STUDY LIMITATIONS

Despite the promising findings, this study has several limitations that must be acknowledged. Firstly, the sample size was relatively small, which may limit the generalizability of our results to a broader population. Additionally, this was a single-center study, potentially introducing selection bias and limiting external validity. The retrospective nature of our analysis further constrains our ability to establish causality between the use of Respin 11 (FP-HFCWO) and the observed clinical improvements. Another important limitation is the relatively short follow-up period for patients undergoing Respin 11 therapy, which prevents us from drawing definitive conclusions regarding its long-term effectiveness and sustainability, and the variable duration of follow-up across patients may have introduced bias in exacerbation rate estimates, as no time-standardized or survival analyses were performed. Additionally, the absence of a control group in this retrospective design inherently limits causal inference. It is acknowledged that seasonal variability in exacerbation rates may have contributed to the reduction observed. While the patient population was continuously enrolled and followed across different months to mitigate seasonal bias, no historical cohort or parallel comparator was available to control for temporal confounders. The generalizability of these findings may be limited by the single-center design and the demographic characteristics of the COPD cohort, which predominantly consisted of elderly patients. These factors may not fully reflect the broader population affected by obstructive lung diseases, particularly younger individuals or those managed in different care settings. Lastly, potential heterogeneity between the COPD and bronchial asthma groups—due to differences in underlying pathophysiology, disease severity, and treatment history—may have influenced the outcomes, necessitating further stratified and prospective studies to refine these findings. Future research with larger, multicenter cohorts and longer follow-up periods will be essential to validate and expand upon these initial observations.

## CONCLUSION

This retrospective cohort study demonstrated that Focal Pulse High-Frequency Chest Wall Oscillation FP-HFCWO therapy (Respin 11, VitalAire®) significantly improved clinical outcomes in patients with obstructive lung diseases (COPD and asthma) presenting with chronic mucus hypersecretion and poor disease control despite a triple treatment, along with mucolytics. FP-HFCWO treatment was associated with substantial reductions in symptom burden, as reflected by improved CAT and ACT scores, and a notable decrease in the frequency of moderate-to-severe exacerbations during the follow-up when compared to the year before Respin 11 therapy.

These findings suggest that FP-HFCWO may serve as an effective adjunctive treatment for optimizing disease management in patients with mucus-related obstructive lung disease. However, given the study’s limitations, including its retrospective design, small sample size, single-center setting, and short follow-up duration, further large-scale, prospective studies are warranted to confirm these results, explore long-term benefits, and assess the broader applicability of this intervention in routine clinical practice.

## Figures and Tables

**Fig. (2):**
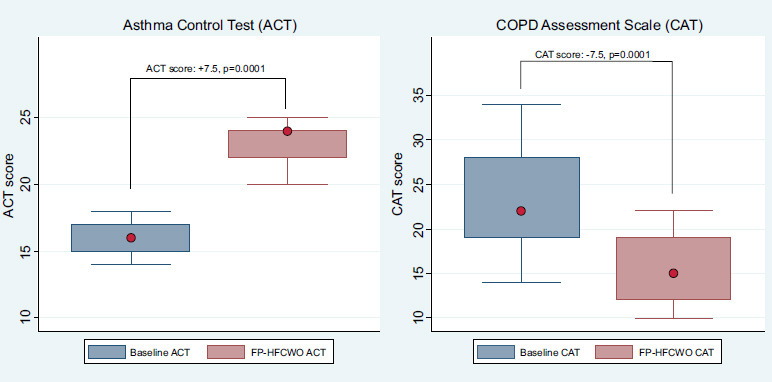
Impact of FP-HFCWO on ACT and CAT scores.

**Fig. (3):**
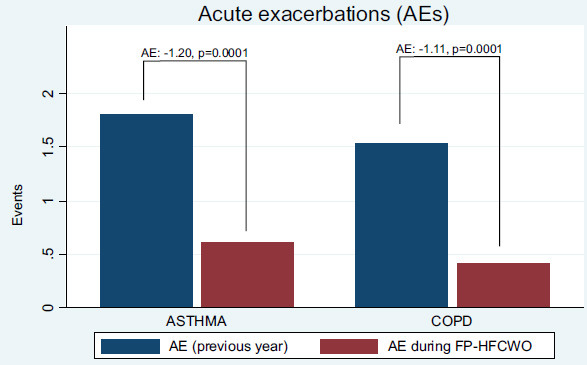
Impact of FP-HFCWO in acute exacerbations of asthma and COPD.

**Table 1:** Dataset and analysis of the COPD / asthma subgroup.

**Variables**	**All pts**	**COPD**	**Asthma**
Age	74.2	78.0	67.8
Respin therapy (time, months)	8.0	7.17	9.30
Fixed ratio (FEV1/FVC)	0.67	0.59	0.81
FEV1%	69.7%	64.7%	78.2%
FVC%	88.9%	87.8%	90.5%
FEF25-75%	47.2%	43.8%	53%
ACT baseline	15.5	n/a	15.5
CAT baseline	23.2	23.2	n/a
HRCT (small airways features)	51.9%	47%	50%
Active smoker (%)	55%	52.9%	60%
Acute exacerbations before FP-HFCWO	1.63	1.52	1.8
Acute exacerbations during FP-HFCWO	0.48	0.41	0.6

**Table 2:** Changes in COPD and asthma during FP-HFCWO treatment.

**Group**	**CAT/ACT Baseline**	**CAT/ACT Post**	**Δ**	***p*-value**	**Exacerbations Before**	**Exacerbations After**	**Δ**	***p*-value**
**COPD**	23.2	15.7	-7.52	0.001	1.53	0.41	-1.11	0.001
**Asthma**	15.5	23.0	+7.50	0.001	1.80	0.60	-1.20	0.001

## Data Availability

The data sets used and analysed during this study are available from the corresponding author [P.V.M] upon request.

## LIST OF ABBREVIATIONS

ACT: = Asthma Control Test
CAT: = COPD Assessment Test
CMH: = Chronic Mucus Hypersecretion
COPD: = Chronic Obstructive Pulmonary Disease
FEF25–75%: = Forced Expiratory Flow at 25–75% of FVC
FEV_1_: = Forced Expiratory Volume in 1 Second
FP-HFCWO: = Focused Pulse High-Frequency Chest Wall Oscillation
FVC: = Forced Vital Capacity
HRCT: = High-Resolution Computed Tomography
ICS: = Inhaled Corticosteroids
LABA: = Long-Acting Beta-Agonist
LAMA: = Long-Acting Muscarinic Antagonist
MCID: = Minimal Clinically Important Difference
NAC: = N-Acetylcysteine
SD: = Standard Deviation
SD: = Standard Error

## STANDARDS OF REPORTING

STROBE guidelines were followed.

## DECLARATIONS

This manuscript has undergone formal and grammatical revision using artificial intelligence tools. AI was not used for data generation, analysis, interpretation, or the formulation of the study’s conclusions.
